# Generative AI in clinical (2020–2025): a mini-review of applications, emerging trends, and clinical challenges

**DOI:** 10.3389/fdgth.2025.1653369

**Published:** 2025-11-03

**Authors:** Nafiz Fahad, Riadul Islam Rabbi, Sumayea Benta Hasan, Fariya Sultana Prity, Rasel Ahmed, Farhana Ahmed, Md. Jakir Hossen, Tze Hui Liew, Md Shohel Sayeed, Kah Ong Michael Goh

**Affiliations:** ^1^ Faculty of Information Science and Technology (FIST), Multimedia University, Melaka, Malaysia; ^2^ Faculty of Engineering and Technology, Multimedia University, Melaka, Malaysia; ^3^ Department of Computer Science, American International University-Bangladesh, Dhaka, Bangladesh; ^4^ Faculty of Science and Technology, American International University-Bangladesh, Dhaka, Bangladesh; ^5^ Center for Advanced Analytics (CAA), COE for Artificial Intelligence, Faculty of Engineering & Technology (FET), Multimedia University, Melaka, Malaysia; ^6^ Centre for Intelligent Cloud Computing (CICC), COE of Advanced Cloud, Faculty of Information Science & Technology, Multimedia University, Melaka, Malaysia; ^7^ Center for Image and Vision Computing, COE for Artificial Intelligence, Faculty of Information Science and Technology, Multimedia University, Melaka, Malaysia

**Keywords:** generative AI, electronic-health-record, GANs, diffusion models, Vision-Language Models

## Abstract

Generative artificial intelligence (G-AI) has moved from proof-of-concept demonstrations to practical tools that augment radiology, dermatology, genetics, drug discovery, and electronic-health-record analysis. This mini-review synthesizes fifteen studies published between 2020 and 2025 that collectively illustrate three dominant trends: data augmentation for imbalanced or privacy-restricted datasets, automation of expert-intensive tasks such as radiology reporting, and generation of new biomedical knowledge ranging from molecular scaffolds to fairness insights. Image-centric work still dominates, with GANs, diffusion models, and Vision-Language Models expanding limited datasets and accelerating diagnosis. Yet narrative (EHR) and molecular design domains are rapidly catching up. Despite demonstrated accuracy gains, recurring challenges persist: synthetic samples may overlook rare pathologies, large multimodal systems can hallucinate clinical facts, and demographic biases can be amplified. Robust validation, interpretability techniques, and governance frameworks therefore, remain essential before G-AI can be safely embedded in routine care.

## Introduction

Healthcare has long grappled with the twin problems of data scarcity and data privacy. Curating large, balanced, and publicly shareable clinical datasets is expensive, logistically complex, and ethically sensitive. Recent advances in generative artificial intelligence (G-AI)—notably Generative Adversarial Networks (GANs), variational auto-encoders, diffusion models, and large Vision-Language Models (VLMs)—offer a potential remedy by synthesising realistic yet privacy-preserving data. Table 1 collates fifteen representative studies that demonstrate how these models are already reshaping diverse clinical tasks.

**Table 1:** Summary of clinical generative AI applications.

Citation	Dataset	Method	Used G. AI	Application of used G. AI	Purpose in healthcare	Limitations of used G. AI
Bhatt et al. (2025) (13)	Medical images, patient records, patient medical histories	Data acquisition, Data preprocessing, Model training (GANs, VAEs, RNNs, DCGAN, DRL), Synthetic data-generation, Evaluation, Application development	Generative Adversarial Networks (GANs) Variational Autoencoders (VAEs) Recurrent Neural Networks (RNNs) Deep Reinforcement Learning (DRL)	Synthetic data generation to enhance medical datasets Improvement in disease diagnosis (e.g., breast cancer, vertebral fractures) Radiology imaging enhancement (x-ray, CT, MRI, PET imaging) Medical education (training simulations with generated scenarios) Drug discovery and personalized treatment plans	Augment limited real-world data to train better machine learning models Improve drug discovery and biomedical instrument design Enhance early diagnosis through realistic synthetic medical imaging Preserve patient privacy by generating synthetic data Assist medical education with risk-free virtual learning scenarios	Data quality and quantity: Healthcare datasets often have noise and missing values Lack of interpretability: Difficult to explain how the AI arrived at the results Ethical and regulatory concerns: Need for strong privacy protections Generalization issues: Risk of overfitting to training data and generating unrealistic results Bias and fairness: Biases in training data can produce unfair outcomes Safety and reliability: Inaccurate outputs can lead to dangerous clinical decisions
Ultsch et al. (2025) (4)	SIIM-ISIC Melanoma Classification Dataset, PH2 Dataset	Fine-tuned a Stable Diffusion model to generate synthetic dermoscopic images for melanoma detection	Stable Diffusion model, which is a latent diffusion model (LDM)	Synthesize realistic dermoscopic images of melanoma benign skin lesions to augment training datasets for classification models	To address data scarcity and class imbalance in melanoma detection datasets improving the performance of AI models in skin cancer diagnosis	Synthetic images may not capture all real-world variations risk of overfitting synthetic features potentially limiting generalizability
Pawlicka et al. (2024) (3)	Colorectal polyp dataset	StyleGAN2 to synthesize polyp images perform segmentation using a model trained on a mix of real and synthetic images	StyleGAN2 Generative Adversarial Network (GAN)	Synthesize realistic polyp images to augment the training dataset improve the performance of downstream polyp segmentation models	Address data scarcity in medical imaging by generating synthetic but realistic data enhance segmentation model performance, which is critical for early and accurate detection of colorectal cancer	Diversity of generated images may still not fully cover real-world variations in polyp appearances risk of distribution mismatch between synthetic and real images, which could potentially affect the generalization of the trained segmentation models
Aydin et al. (2024) (2)	Utilized Time-of-Flight Magnetic Resonance Angiography (TOF MRA) scans sourced from six open-source datasets	StyleGANv2 architecture to a 3D format specifically for generating synthetic TOF MRA volumes	3D version of StyleGANv2, which is a type of Generative Adversarial Network (GAN)	Enhance the training of deep learning models for multiclass semantic segmentation of CoW arteries improve the performance of segmentation tasks, which are critical for diagnosing and treating cerebrovascular diseases	To address the limitations of real patient data, such as scarcity, high costs, and regulatory challenges to provide a more diverse and representative dataset that can improve the performance of deep learning models in medical imaging	The synthetic data produced may have limited anatomical fidelity or downstream utility in specific tasks related to vessel characteristics
Khosravi et al. (2024) (9)	Pelvic radiographs from patients undergoing total hip arthroplasty	generative deep learning (DL) technology specifically using denoising diffusion probabilistic models	denoising diffusion probabilistic model	visualize and analyze race-based disparities within large imaging registries identify and characterize systematic differences in radiographs between African American and White patients	enhance understanding of underlying differences in medical imaging datasets identify biases in downstream tasks, ultimately fostering the development of fairer healthcare practices and improving patient care	reliance on self-reported race, which may not capture the full spectrum of patient diversity dataset's demographic composition may limit the generalizability of the findings to other healthcare settings improvement in generating diagnostic-grade images and validating findings in independent datasets
Patel et al. (2024) (14)	Facial images with genetic conditions	DL image classifier HyperStyle, a GAN-inversion technique compatible with StyleGAN2	HyperStyle technique, which allowed for the alteration of facial expressions in the images while maintaining phenotypic accuracy	Enhance diagnostic accuracy in medical genetic understand how changed expressions could affect clinicians’ ability to diagnose genetic conditions quicker and more cost-effective diagnoses, especially in underserved communities	Diagnostic process for genetic conditions provide valuable insights that would assist clinicians in making more informed decisions	While generative AI can be beneficial, it is crucial to identify and mitigate confounding factors that may impact the results, particularly in clinical applications
Lang et al. (2024) (12)	Retinal fundus photographs external eye photographs chest radiographs	Training a classifier on the image dataset training a StyleGAN-based image generator called “StylEx,” automatically detecting and visualizing top visual attributes formulating hypotheses based on these attributes	StyleGAN architecture, specifically the StylEx model	Identify and visualize discrete medical imaging features that correlate with demographic information and systemic conditions uncover new insights that may not be readily identifiable by human experts, thereby enhancing the understanding of AI models in healthcare	Improve the explainability of AI models in medical imaging facilitate hypothesis generation and enhance the understanding of the underlying mechanisms that link visual changes to health outcomes	Not designed to infer causality real-world biases and socio-cultural factors could complicate the interpretation of results and necessitate careful consideration by interdisciplinary experts
Alkhalaf et al. (2024) (11)	Electronic health records (EHRs) related to malnutrition management	Llama 2 13B model with a zero-shot prompting technique Retrieval Augmented Generation (RAG)	Llama 2 an open-source model	Summarize clinical notes and extracting key information about malnutrition risk factors from EHRs generate structured summaries about clients’ nutritional status and identifying risk factors for malnutrition	Efficiently extract key clinical information from large volumes of EHR data improve understanding of malnutrition issues and facilitate the development of effective interventions in aged care settings	Model hallucination, where the AI generates plausible but unverified outputs
Pinaya et al. (2023) (10)	Chestx-ray14	Chest x-ray Pathology Synthesis (CXP-Syn) an approach combining conditional denoising diffusion probabilistic models (DDPMs)	Denoising Diffusion Probabilistic Model (DDPM)	Synthesize realistic and diverse chest x-ray images data augmentation to improve downstream tasks like disease classification	Augment limited datasets in medical imaging improve diagnostic model performance assist radiologists and developers	May not capture extremely rare or subtle pathologies risk of generating unrealistic combinations of abnormalities Potential biases in the Chestx-ray14 dataset
Bordukova et al. (2023) (8)	Patient-derived data includes baseline measurements and prior clinical trajectories	Machine learning (ML) techniques	Novel, realistic, and complex data with desired properties for developing Digital Twins (DTs) in drug discovery and clinical trials	Create Digital Twins, which are digital replicas of physical systems enhancing the efficiency of drug discovery and development	Increase the efficiency of drug discovery and development processes digitalizing processes that are typically associated with high economic, ethical, or social burdens, ultimately advancing precision medicine	Current state of Digital Twins in drug discovery does not fully exploit the potential of generative AI
Huang et al. (2023) (6)	Randomly sampled emergency department encounters at a tertiary care institution	Retrospective diagnostic study where an AI interpretation was generated for each chest radiograph	Multimodal generative artificial intelligence methodologies	Create chest radiograph reports in the emergency department (ED) setting	Optimize emergency department care by providing near-instant interpretations of medical imaging supports high case volumes and aids in clinical decision-making	Challenge of objectively evaluating the accuracy of free-text imaging interpretations
La Salvia et al. (2022) (15)	Synthetic hyperspectral medical images, specifically targeting epidermal lesions related to skin cancer	Deep Convolutional Generative Adversarial Network (DCGAN)	DCGAN, which is a type of generative adversarial network specifically designed for generating high-quality images	Skin cancer diagnosis provide a robust dataset that can be used to train deep learning classifiers, thereby enhancing diagnostic capabilities in healthcare	Overcome the challenges posed by small-sized datasets in healthcare facilitate the training of deep learning models, which can lead to improved diagnostic tools and surgical guidance in clinical practice	Need for researchers to provide knowledge regarding the distribution of synthetic and original data
Zeng et al. (2022) (7)	Does not specify a particular dataset	Hierarchical generative models and ProteinGAN, which incorporates a self-attention mechanism	Deep generative models	Design small molecules and proteins with desired therapeutic properties for drug discovery and development	Accelerate drug discovery processes by generating novel compounds and therapeutic proteins	Often capture only shallow statistical correlations which leads to misleading decisions
Han et al. (2020) (1)	Annotated medical images collected for Computer-Aided Diagnosis (CAD) research	Generative Adversarial Networks (GANs)	Pathology-aware Generative Adversarial Network (GAN)	Educate physicians who may not have extensive experience in interpreting complex medical images aiding healthcare professionals in making more informed decisions based on enhanced image data	Bridge the gap between AI and healthcare by providing clinically relevant tools that can improve diagnostic processes confirm the clinical relevance of the GAN-based image augmentation techniques	Need to confirm the clinical relevance of the generated images for diagnosis
Liu,F er al (2025) (16)	8M EHRs (14.8B tokens) 5.4M academic articles (48B tokens) 15,731 medical textbooks (8.6B tokens)—630k rare disease/emergency EHRs 600k chest x-rays (CXR) 24k CT scans QA datasets (PubMedQA, MedQA, MedMCQA) OpenAssistant Conversations, CoT Collection	Base: Qwen-1.5 32B Pretraining on multimodal structured + unstructured data Multimodal instruction fine-tuning Evaluation via manual physician grading + automated metrics (accuracy, F1, precision, recall, ROUGE-L, BertScore)	MetaGP (Meta General Practitioner) — 32B parameter generative foundation model for medicine	Rare disease diagnosis Emergency condition identification Clinical decision support Radiology report generation (CXR, CT) Multimodal data integration (EHR + imaging + QA tasks)	Improve diagnostic accuracy across rare/emergent cases Enhance physician performance (esp. juniors) Generate reliable imaging reports comparable to radiologists Reduce harmful outputs and bias risks	Model size smaller than GPT-4 may limit retention of broad medical knowledge Transparency/interpretability challenges in decision-making Ethical risks: over-reliance, bias, fairness concerns Heavy computational resource demands (A100 GPUs)

^a^ Limitations were reported as stated in the original studies where available; otherwise, we added interpretive remarks to highlight potential concerns (marked with †). This distinction helps clarify which insights stem directly from prior work vs. our critical synthesis†^a^.

Medical imaging remains the most prolific test-bed for G-AI. Early work by Han et al. introduced “pathology-aware” GANs that augment computer-aided-diagnosis (CAD) datasets and serve as training material for novice radiologists (1). Subsequent studies refined both fidelity and dimensionality of synthetic images. Aydin et al. re-engineered StyleGANv2 to generate three-dimensional Time-of-Flight MR angiography volumes, boosting multiclass artery segmentation without additional patient scans (2). Similar philosophies underpin Pawlicka et al.'s colorectal-polyp synthesis, where GAN-generated images alleviate class imbalance and improve endoscopic segmentation accuracy (3). Ultsch and Lötsch addressed melanoma detection by fine-tuning a latent Stable Diffusion model, proving that diffusion-based methods can rival GANs for dermoscopic realism (4).

The promise of G-AI is not limited to raw pixels. Phipps et al. explored VLMs that translate chest x-ray features into free-text radiology reports, potentially reducing radiologist workload during high-volume shifts (5). However, their evaluation framework also revealed a tendency to hallucinate clinical findings—a stark reminder that factual grounding remains a critical bottleneck. Complementary efforts by Huang et al. in emergency-department workflows corroborate both the efficiency gains and the evaluation challenges of text-generating models (6).

Beyond imaging, G-AI is venturing into molecular and systemic domains. Zeng et al. leveraged ProteinGAN and hierarchical generative models to design novel proteins and small molecules, accelerating the pre-clinical discovery pipeline (7). Bordukova et al. harnessed synthetic patient trajectories to construct digital twins that can de-risk costly clinical trials (8). At the intersection of fairness and analytics, Khosravi et al. generated radiographs that isolate race-linked imaging features, providing a sandbox for bias audits (9).

These successes nonetheless surface persistent limitations. Synthetic data often fails to capture rare anatomical variants or subtle disease phenotypes, risking model over-confidence in out-of-distribution scenarios (4, 10). Bias in training corpora can be magnified, as evidenced by demographic skew in pelvic-radiograph synthesis (9). Large multimodal systems may produce credible but incorrect statements, undermining clinical trust (5, 11). Interpretable frameworks such as StylEx, which links StyleGAN latents to human-readable attributes, are therefore gaining traction (12).

Regulatory and ethical considerations further complicate deployment. Frictionless data-sharing enabled by G-AI must still honor patient consent and institutional review protocols. Meanwhile, explainability demands are intensifying; clinicians and regulators alike now expect transparent reasoning pathways before sanctioning AI-assisted decisions. Collectively, the studies surveyed here illuminate both the transformative potential of G-AI and the rigorous safeguards required for its responsible translation to bedside practice.

## Methodology of literature selection

To identify relevant studies, we conducted a targeted search in PubMed, IEEE Xplore, and Scopus databases covering January 2020–May 2025. Keywords included “generative AI”, “synthetic data”, “clinical practice”, and “healthcare”. From over 65 initial hits, we prioritised peer-reviewed articles that explicitly applied generative AI in clinical contexts. Fifteen representative studies were chosen to illustrate diverse domains (imaging, text, molecular design, and fairness). These were not intended as an exhaustive list, but rather as exemplars highlighting the breadth and key limitations of generative AI in healthcare.

## Comparative analysis and discussion

Table 1 distills fifteen recent studies that deploy generative AI (G-AI) across the clinical data spectrum, with medical imaging emerging as the prime test-bed. More than two-thirds of the entries apply GANs, diffusion models or Vision-Language Models (VLMs) to synthesize, augment or interpret radiographs, MRI volumes and dermoscopic, endoscopic or fundus photographs. These image-centric efforts tackle three chronic bottlenecks highlighted in Table 1: limited data volume, class imbalance and privacy restrictions. For example, Ultsch & Lötsch fine-tune Stable Diffusion to balance melanoma classes, while Aydin et al. extend StyleGANv2 to 3-D angiography volumes, boosting vascular-segmentation accuracy without collecting new scans.

Beyond imaging, Table 1 shows G-AI penetrating narrative and molecular domains. Alkhalaf et al. couple a retrieval-augmented Llama-2 with zero-shot prompting to summarise malnutrition risk factors from electronic health records, illustrating how foundation models can tame unstructured clinical text. Zeng et al. harness ProteinGAN to generate bespoke proteins, signalling a shift from data augmentation to de-novo biomedical design. Meanwhile, Pinaya and Bordukova exploit diffusion models to create synthetic chest x-rays and digital-twin trajectories respectively, lowering the cost and ethical burden of large-scale trials.

The table also exposes recurring limitations. Synthetic samples often omit rare pathologies, risk distribution shifts (e.g., Pawlicka's colorectal polyps) or encode demographic biases (Khosravi's race-aware radiographs). VLMs hallucinate clinical facts, undermining trust in auto-generated reports. Several authors therefore call for stronger interpretability—Lang's StylEx explicitly pairs StyleGAN with attribute visualisation—and for rigorous external validation before clinical rollout.

Collectively, the evidence in Table 1 suggests three near-term pay-offs: (i) privacy-preserving data augmentation that accelerates model development, (ii) automation of expert-intensive tasks such as radiology reporting or phenotype annotation, and (iii) exploratory insight generation that surfaces novel biomarkers or inequities. Realising these benefits, however, hinges on closing interpretability gaps, curbing bias propagation, and establishing governance frameworks that keep pace with rapidly evolving G-AI toolchains. To mitigate these concerns, safeguards such as bias audits, explainability techniques, and transparent provenance tracking of synthetic data should be incorporated into deployment frameworks. Evaluation of generative models is often benchmarked with metrics such as BLEU/ROUGE for text, Fréchet Inception Distance (FID) or Inception Score for images, and perplexity for language models, which provide quantitative grounding for reliability assessments.

## Conclusion

Generative AI is already enriching clinical data pipelines, from radiology suites to drug-discovery labs. The reviewed literature confirms tangible gains in diagnostic accuracy, workflow efficiency, and hypothesis generation, driven chiefly by image-focused GANs, diffusion models, and emerging VLMs. Yet every advantage is tempered by unresolved issues of bias, fidelity, and interpretability. Rare pathologies remain under-represented, demographic disparities can be inadvertently reinforced, and text generators are prone to clinically dangerous hallucinations. Future work must therefore pair technical innovation with stringent validation on external cohorts, transparent reporting of synthetic-data provenance, and user-friendly explanation interfaces. Only through such multidisciplinary vigilance can G-AI move from promising prototypes to trustworthy, equity-focused tools that genuinely advance patient care. Emerging trends such as text-to-3D generation for surgical planning signal new directions for generative AI in clinical practice, while broader applications in education and management remain outside the scope of this review.
